# Insights of roles played by septins in pathogenic fungi

**DOI:** 10.1080/21505594.2021.1933370

**Published:** 2021-06-07

**Authors:** Lin Li, Xue-Ming Zhu, Zhen-Zhu Su, Maurizio Del Poeta, Xiao-Hong Liu, Fu-Cheng Lin

**Affiliations:** a State Key Laboratory for Managing Biotic and Chemical Treats to the Quality and Safety of Agro-products, Institute of Plant Protection and Microbiology, Zhejiang Academy of Agricultural Sciences, Hangzhou, China; b State Key Laboratory for Managing Biotic and Chemical Treats to the Quality and Safety of Agro-products, Institute of Biotechnology, Zhejiang University, Hangzhou, China; c Department of Microbiology and Immunology, Stony Brook University, Stony Brook, New York, USA; d Division of Infectious Diseases, Stony Brook University, Stony Brook, New York, USA; e Veterans Affairs Medical Center, Northport, New York, USA

**Keywords:** Septins, pathogenic fungi, biological functions, pathogenesis

## Abstract

Septins, a conserved family of GTP-binding proteins, are widely recognized as an essential cytoskeletal component, playing important roles in a variety of biological processes, including division, polarity, and membrane remodeling, in different eukaryotes. Although the roles played by septins were identified in the model organism *Saccharomyces cerevisiae*, their importance in other fungi, especially pathogenic fungi, have recently been determined. In this review, we summarize the functions of septins in pathogenic fungi in the cell cycle, autophagy, endocytosis and invasion host-microbe interactions that were reported in the last two years in the field of septin cell biology. These new discoveries may be expanded to investigate the functions of septin proteins in fungal pathogenesis and may be of wide interest to the readers of Microbiology and Molecular Pathology.

## Introduction

**Figure 1.**
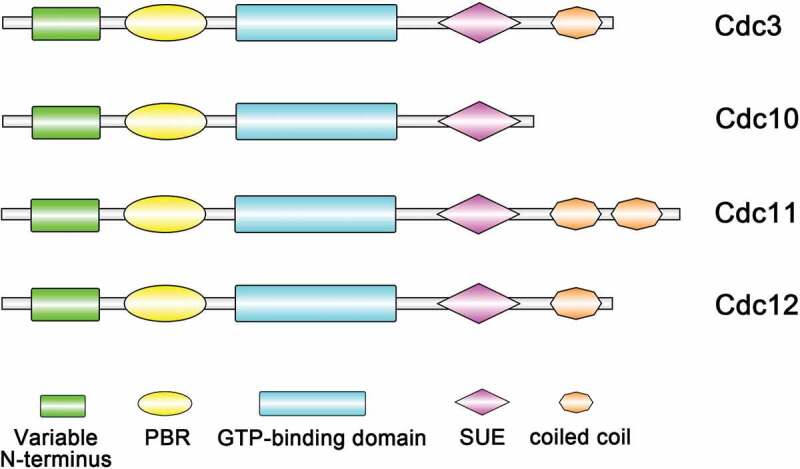
Domain structures of *S. cerevisiae* core septins. All septins contain a GTP-binding domain, the septin unique element (SUE) and a phosphoinositide-binding polybasic region (PBR). The C-terminus (coiled-coil) regions vary

Septins are a family of ﬁlament-forming GTP-binding proteins that were first identified in *Saccharomyces cerevisiae*, functioning in many eukaryotic cells and playing vital roles throughout life [1–3]. Research has found that septins could form the ring of filaments located at the mother-bud neck [4], where the septum or cell wall separating the mother and daughter cell is found, and thus, they were named “septins”. Septins are GTP-binding proteins containing a P-loop and are variable at both the N-terminus and the C-terminus. In many species, septins have a highly conserved central core, consisting of a N-terminus polybasic domain that has been shown to bind to phosphoinositide on the plasma membrane [5,6]. Downstream is the GTP-binding domain, which ends with a unique septin element that distinguishes septins from other members of the P-loop-containing GTPase family and a specific 53 amino-acid stretch called the septin unique element (SUE) [7,8]. The variable C-terminus after the GTPase domain can contain zero, one, two or three coiled coil domains (Figure 1) [5,7,9,10].

**Figure 2.**
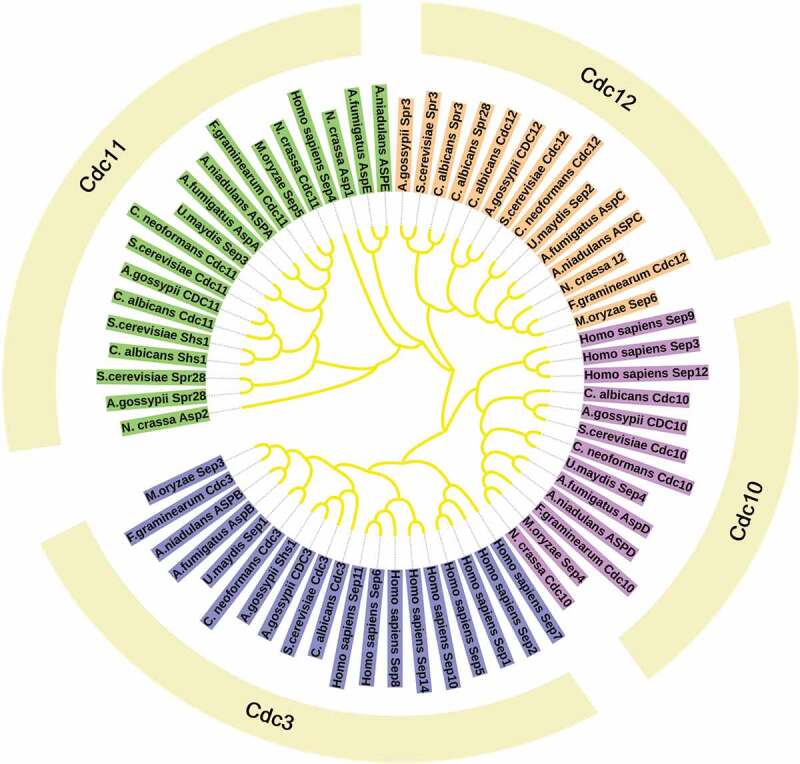
Phylogeny and evolutionary history of septins. Maximum likelihood phylogenetic tree of 65 septin proteins from 11 fully sequenced species that are reported in eukaryotic lineages. The categorization of different septins by multiple copies of coiled coils found in a single protein: the Cdc11 group with two coiled-coil domains, the Cdc3 and Cdc12 groups with one coiled-coil domain and the Cdc10 group with no coiled-coil domain

Previous studies have classically described septins to participate in cytokinesis, however, the new functions of septins were found in the last decades and include their roles in other important intracellular processes including acting as scaffolds to recruit binding partners, functioning as diffusion barriers to compartmentalize membrane proteins and even participating in mechanotransduction and plant invasion by fungal pathogens. Although some functions are not as well-characterized as in yeast, septins functions have been recognized in a wide range of eukaryotic species, such as animals and other fungi [7]. In addition, the number of septin varies in different organisms [11]. In *Caenorhabditis elegans*, there are only two septins (UNC-59 and UNC-61) [12]. *S. cerevisiae* encodes seven septins, including five core septins (Cdc3, Cdc10, Cdc11, Cdc12, and Sep7/Shs1) and two specific septins (Spr3 and Spr28) [7]. The plant pathogenic fungi, *Magnaporthe oryzae* contains four yeast septin orthologues and one ancestral septin AspE [13]. *Fusarium graminearum* contains seven putative septin-encoding genes, Cdc3, Cdc10, Cdc11, Cdc12, AspE, AspE2 and Hyp7. Among these, four septins, Cdc3, Cdc10, Cdc11, Cdc12 are high orthologues of the *S. cerevisiae* septins and called “core” septins [7]. *Cryptococcus neoformans* and *Ustilago maydis* can only express core septins (Table 1) (Figure 2) [14,15].

**Table 1.** Septins and their functions in fungi

Organism	Mutants	Virulence	Function	References
*S. cerevisiae*	Δ*cdc3*	NI	Cell cycle regulation, Cell morphogenesis, Diffusion barrier, Vesicle trafficking, Microtubule stability, Cortical rigidity, Bud site selection, Autophagy progress	[1,25,51,103,104]
	Δ*cdc10*			
	Δ*cdc11*			
	Δ*cdc12*			
	Δ*shs1*			
	Δ*spr3*			
	Δ*spr28*			
*M. oryzae*	Δ*Mosep3*	Reduced virulence on rice	Septation, Invasion growth, Appressorium formation, Cell cycle checkpoint, Autophagy, Pathogenicity	[13,21,22,43,44]
	Δ*Mosep4*			
	Δ*Mosep5*			
	Δ*Mosep6*			
*F. graminearum*	Δ*Fgcdc3*	Reduced virulence on wheat	Septation, Nuclear division, Conidiation, Stress response, Ascospore formation, Sexual reproduction, Virulence	[86]
	Δ*Fgcdc10*	Virulent on wheat		
	Δ*Fgcdc11*	Reduced virulence on wheat		
	Δ*Fgcdc12*	Reduced virulence on wheat		
*A. nidulans*	Δ*aspA*	NI	Septation, Nuclear division, Conidiation, Negative regulation of new grow foci	[45,46,105,106]
	Δ*aspB*			
	Δ*aspC*			
	Δ*aspD*			
	Δ*aspE*			
*A. fumigatus*	Δ*aspA*	Hypervirulent in *Galleria mellonell*	Cell morphology, Ascospore formation, Cell division site (separation)	[90]
	Δ*aspB*			
	Δ*aspC*			
	Δ*aspD*			
	Δ*aspE*			
*U. maydis*	*sep1*∆	Reduced virulence on corn	Morphogenesis, Filamentous growth, Teliospore formation	[14,86]
	*sep2*∆			
	*sep3*∆			
	*sep4*∆			
*C. neoformans*	*cdc3*Δ	Reduced virulence in *Galleria*	Sexual reproduction, Nuclei distribution, Cell wall stress response, Pathogenicity	[15]
	*cdc10*Δ	NI		
	*cdc11*Δ			
	*cdc12*Δ

**Figure 3.**
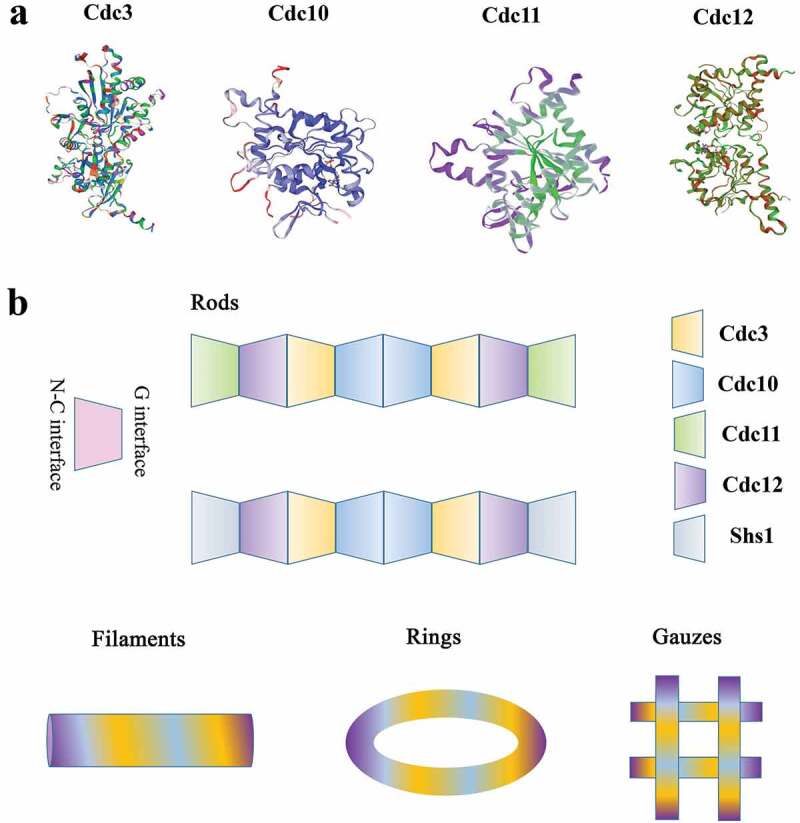
Structures of the septins. (a) The predicted structures of different septins in yeast cells. (b) Schematic diagram of the yeast septin rod, filament, ring, and gauze. The yeast hetero-octameric septin complex is a linear rod with the subunits arraying in the order and with the interfaces indicated. Each septin forms associations with its neighbors through either a G interface or an N-C interface. The rod septins can form high-order structures, like filaments, rings, gauzes

Recent discoveries have shown that septins were built to function as complexes rather than single protein entities and form complex septin ultrastructures, such as filaments, rings and gauzes [16]. How do these different structures form and what roles do these different structures play? In *S. cerevisiae*, the molecular structure of the septin complex was solved by using single-particle electron microscopy and proposed that Cdc10 occupies the central position of the core complex and Cdc11 lies at its extremities, leading to the following arrangement for the octamer: Cdc11-Cdc12-Cdc3-Cdc10-Cdc10-Cdc3-Cdc12-Cdc11. The linear rod lacks polarity. When the salt ion concentration is low, the N-C interface of Cdc11 in the rod is connected to form filaments. In addition, the predicted C-terminal coiled-coil domains of Cdc3 and Cdc12 are required to assemble into filaments [17]. In *S. cerevisiae*, septins comprise another hetero-octamer: Shs1-Cdc12-Cdc3-Cdc10-Cdc10-Cdc3-Cdc12-Shs1. The GTPase activity of Cdc12 determines that Cdc11 or Shs1 is located at the end of the octamer. Recent studies have shown that when the guanidinium binds to septin at the site where arginine exist, septin proteins assemble non-native Cdc11/Shs1-Cdc12-Cdc3-Cdc3-Cdc12-Cdc11/Shs1 hexamers [18]. The filament structures would change to ring and gauze-like structures when the cell wall is removed through enzymatic digestion (Figure 3) [19]. These results suggest that septins function as complexs rather than as single protein entities. The ring structures appeared at the mother-bud neck in yeast [20] and the appressorium-penetration peg neck in rice blast fungus associated with the curved membrane and virulence [21]. New research demonstrates that septin proteins bind phosphatidylinositol phosphates (pips) at the appressorium pore membrane to assemble into a ring, promoting the formation of a penetration peg required for host infection in *M. oryzae* [22]. In addition to filaments and rings, septins can also form structures referred to as gauzes, which consist of a meshwork of septin filaments in yeast [23]. Through immunoelectron microscopy, it was found that Cdc3 was mainly localized to the gauze structure. The temperature-sensitive allele of Cdc12 is an essential septin for the yeast septin complex, and it lacks the gauzes structure when grown at nonpermissive temperatures. All in all, these results indicate that gauzes are composed of septins (Figure 3b) [19].

Numerous functions of septins have been explored, such as in cytokinesis, cell cycle control, spatial compartmentalization, exocytosis and as a barrier to endoplasmic reticulum (ER) and plasma membrane diffusion [16,24]. However, the roles of septin complexes in pathogenic fungi and how they contribute to host infection are still largely unknown. Currently, new evidences have shown that septins promote the formation of autophagosomes and endosomes by recruiting Atgs [25]. These researches suggested that septins may be have new functions that involved in growth and pathogenesis of fungi. In this review, we discuss the biological functions of septins in pathogenic fungi based on recent research.

## Septin function is linked to the assembly of ﬁlaments and is involved in the cell cycle

A key aspect of septin function is that septin ring formation is regulated during the cell cycle by assembly and disassembly [26–28]. Five septins (Cdc3, Cdc10, Cdc11, Cdc12 and Shs1) are recruited to the presumed bud side in a way that depends on Cdc42 after yeast enters a new cell cycle [29]. It has been reported that many septins and colocalize with microtubules at different cell cycle stages in many model organisms and various mammalian cell types [30]. Septins remain at the bud neck throughout division and are implicated in bud-site specification, bud growth, nuclear positioning, and cell cycle checkpoint regulation [6,31]. Bud morphogenesis and normal cell cycle progress require the Swe1 protein kinase, which phosphorylates the cyclin-dependent kinase Cdc28, causing G2 arrest [32]. Swe1 is localized to the nucleus and to the daughter side of the bud neck [33]. A family of Nim1-related kinases promote Swe1 localization to the bud neck for degradation and cause progression of the cell cycle [34,35]. All Nim1-related kinases localize to the bud neck in a septin-dependent manner [32,36]. Gin4 and Kcc4, which are synergistic with PAK kinase Cla4, are necessary for septin reorganization. When the septin cytoskeleton is properly organized, Hsl1 is activated by Cdc11 and Cdc12 [36]. Activated Hsl1 recruits Hsl7 to the bud neck [37]. The activation of both Hsl1 and Hsl7 recruits Swe1 kinase to the bud neck, where it is highly phosphorylated, inactivated and finally degraded to enter mitosis. In septin mutant cells or in the case of abnormal morphology, Hsl1 and Hsl7 are released from the bud neck. This causes Swe1 to stabilize and arrest the cell cycle in G2 [32]. This indicates that septin prevents the degradation of Swe1 and thus lead to the arrest of cell cycle.

**Figure 4.**
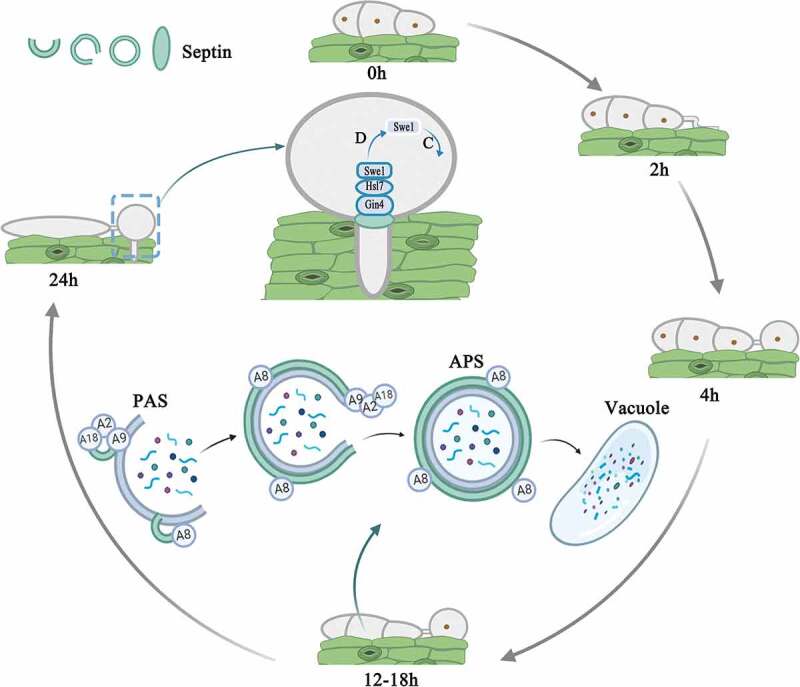
Model showing that septins control conidia autophagy degradation and participate in the development of the penetration peg in *Magnaporthe oryzae* by analogy with *Saccharomyces cerevisiae*. Autophagy begins after conidia are induced on the hydrophobic membrane for 12 hours. Under the induction of starvation, septin colocalized with ATG8 (A8) and ATG9 (A9) at the PAS site. Septins are involved in the biosynthesis of autophagosomes. The atypical ring formed by septins is approximately the same diameter as the autophagosome ring in *M. oryzae*. During the formation of the penetration peg 24 hours after induction, septins aggregated in the appressorium pores, recruited Gin4, activated Gin4 recruited Hsl7, Hsl7 recruited Swe1, and Swe1 was subsequently highly phosphorylated and degraded, and the appressorium entered the mitotic progress. (D represents degradation; C represents cell cycle)

In *M. oryzae*, when the three-celled conidium germinate on the surface of rice leaves, a highly polarized germ tube is formed. The germ tube will elongate and flatten, and then the first round of mitosis will occur, with a daughter nucleus migrating to the tip of the enlarged germ tube [38]. Then, a dome-shaped appressorium is formed at the tip of the germ tube. A thick layer of melanin is formed on the cell wall of the appressorium, and glycerol accumulates rapidly in the appressorium. At the same time, the conidium collapses, its nucleus is degraded by autophagy, and the entire content of the conidium is transported to the appressorium [38]. These coupling processes produce huge turgor in the appressorium, up to 8.0 MPa [39,40]. When the maximum turgor is reached, the actin cytoskeleton in the appressorium will dynamically remodel to form a toroidal F-actin network at the base of the cell. Septin GTPases scaffold the cortical F-actin to the appressorium pores, so that rigid penetration peg to rupture the leaf [13]. This process also requires the Nox2 NADPH oxidase complex, which regulates septin assembly and F-actin remodeling so that it can infect plants [40]. Only after the appearance of the penetration peg does the second round of mitosis proceed. Talbot et al. performed live-cell imaging of the actin cytoskeleton during the maturation of the appressorium by expressing actin binding protein gene fusion Lifeact-RFP, and found that there is a F-actin ring at the base of the infection cell surrounding the appressorium pore, which promote the penetration peg to penetrate the leaf. At the same time, they found that septins will form a 5.9-μm ring, which co-localizes with the F-actin network at the appresoorium pore. When the Septin3-6 or Cdc42 were lost, the F-actin were mislocalization and cannot form normal ring structures so that the pathogenicity of the septin mutant is reduced. What’s more, multiple rounds of nuclear division took place during appressorium development in septin mutants [13]. Recent research showed that plant infection by *M. oryzae* requires two independent S-phase cell-cycle checkpoints. The second checkpoint regulates septin-dependent NADPH oxidase-regulated F-actin dynamics to organize the appressorium pore and facilitate the entry of the fungus into host tissue [41]. Osés-Ruiz et al. speculated that turgor generation, cytoskeletal reorganization and cell cycle progression are collectively required for appressorium-mediated plant penetration [42]. The master regulator of these processes is likely to be the Swe1 kinase [42]. Δ*Moswe1* mutants were recently obtained in our lab, and studies have found that MoSwe1 affects the cell cycle and pathogenicity of *M. oryzae*. In addition, MoSep5-GFP localization was abnormal and the normal ring could not be formed at the appressorium pore, which indicated that the abnormal mitosis would affect the localization of septin (unpublished data). These results indicated that septins are involved in the cell cycle of *M. oryzae* (Figure 4). In addition, MoSmo1 is a GTPase-activating protein that interacts with septins (MoSep3, MoSep4, MoSep5 and MoSep6) in *M. oryzae*. MoSmo1 also regulates septin-mediated F-actin organization [43]. NADPH oxidase is involved in septin-mediated plant infection by *M. oryzae* [44]. F-actin tissue requires NADPH oxidase (Nox1 and Nox2) and its regulatory subunit NoxR in appressorium pores, while Nox2 and NoxR are sufficient for septin ring assembly [44]. In *A. nidulans*, temperature-sensitive AspB mutants show hyperbranching. When transferred to a limiting temperature, septins may also recruit components that regulate the induction of mitotic events [45]. AspB temperature-sensitive mutants show a lack of metulae formation, and AspB may act as a scaffold or barrier [46].

## Septins involved in exocytosis and endocytosis pathways

Endocytosis and exocytosis are processes by which cells move substances into or out of cells, which are too large to pass directly through the lipid bilayer of the cell membrane. Endocytosis is the process by which cells engulf external materials into cells through vesicles. Exocytosis is the process by which cells transfer materials from inside to the outside of the cell. Septins interact with exocytic machinery components. Septins can regulate the transport of vesicles to the fusion site of the plasma membrane by interaction with components of the exocyst complex in mammalian cells [47]. Some of the proteins that interact with septins are essential for synaptic vesicle transport and membrane fusion [47]. Septins can control ion channels, their clustering, and the release of synaptic vesicles. The interaction of septins and syntaxin regulates vesicle dynamics [48]. Fung et al. speculated that septins may have two roles in exocytosis. One is to regulate the transfer of vesicles to the fusion site of the plasma membrane by interacting with the components of the extracapsular complex. The other is to act as an interacting partner of syntaxin that controls membrane fusion events [16]. In mouse cells, Septin2 interacted with β-synuclein, NSF (N-ethylmaleimide-sensitive factor) and Hsc70 (heat shock cognate 71 kDa protein). The knockdown of Septin2 or disruption of septin assembly/disassembly impairs interactions between exocytic proteins and inhibits the late steps of exocytosis. Therefore, septin dynamics are essential for exocytosis [49]. In *Candida albicans*, the association of the septin Cdc3 with the exocyst subunits Sec3 and Sec5 was detected by coimmunoprecipitation experiments. The role of septin-exocyst interactions in polarized exocytosis can be further investigated through *C. albicans* hyphal growth [50]. In yeast, the identities of mother and daughter cells begin to diverge at bud emergence separated by a septin ring, suggesting exocytosis is critically important for the septin ring formation. Polarized exocytosis creates a hole in the accumulating septin density, this initial ring creates a clear boundary, restricting the exocytosis strictly around it, thus clearly delineating the daughter cell [51]. In *M. oryzae*, the exocyst complex localizes at the tip of growing hyphae during vegetative growth and is necessary for polarized exocytosis. Organization of the exocyst complex (Sec3, Sec5, Sec6, Sec8, Sec15, Exo70 and Exo84) at the appressorium pore is a septin-dependent process. The septin-mediated assembly of the exocyst is necessary for appressorium repolarization and host cell invasion [21]. These results suggest that septins have vital roles in exocytosis and endocytosis pathways.

## Roles of septins in autophagy

Autophagy is an evolutionarily conserved intracellular waste treatment and recycling process that involves the formation of double-membrane vesicles called autophagosomes that engulf the intracellular material destined for degradation [52,53]. Many stimuli can trigger autophagy, and autophagy caused by insufficient nutrition or stimulation of specific degradation targets is mainly studied in depth. During periods of nutrient deprivation, this self-eating process is essential for cell survival [54,55]. Therefore, autophagy plays a vital role in maintaining cell homeostasis by turning over functionally abnormal intracellular proteins and promoting recovery. In addition, the disruption of this pathway is associated with an increasing number of human diseases, including cancer and neurodegenerative diseases [56–58]. In filamentous fungi, autophagy also plays an extremely important function. *M. oryzae* is a model organism in filamentous fungi. As early as more than ten years ago, Lin Fucheng’s laboratory first discovered autophagy in *M. oryzae*. After knocking out the autophagy gene MoAtg1, autophagy was blocked, and the pathogenicity of the Δ*Moatg1* mutant was lost, indicating that autophagy affects the pathogenicity of *M. oryzae* [59]. Subsequent studies have found that after knocking out MoAtg2-MoAtg10, MoAtg12, MoAtg14-MoAtg16, and MoAtg18 in *M. oryzae*, autophagy is blocked and the pathogenicity is weakened [60–66].

The cellular cytoskeleton is a large, highly dynamic cellular scaffold that has a crucial role in multiple processes, several of which involve membrane rearrangements and vesicle-mediated events [67]. Actin, microtubules, intermediate filaments, and septins are four main cytoskeletal components of cells [68]. Relatively little is known about the roles of the cytoskeleton network in autophagy. Nevertheless, some recent studies have revealed the importance of cytoskeletal elements, such as actin, microtubules and septins, in specific aspects of autophagy [69]. Approximately 30 years ago, the role of actin in autophagy was first discovered. Starved cells failed to produce autophagosomes when treated with actin depolymerizing agents such as cytochalasin D and latrunculin B [70]. Recent studies demonstrated the colocalization of actin filaments with important autophagy markers [71,72]. Moreover, the knockout of Atg7 in mice inhibited the formation of autophagosomes and showed a serious defect in actin assembly because the expression of proteins involved in controlling actin motility is reduced [73]. Actin-related protein 2/3 (Arp2/3) complex plays a role in shaping the autophagosome membrane and in transporting autophagosomes after detachment [69]. Microtubule dynamics are also related to autophagy [74]. Starvation-induced autophagosome formation seems to require a subset of unstable microtubules [75], and the movement of mature autophagosomes may require stable microtubules before fusion with lysosomes [69,74–79]. However, it is poorly understood how septins are involved in the autophagy process. The earliest research on the relationship between autophagy and septins was in bacterial pathogens. Studies have found that some bacterial pathogens have mechanisms to avoid or use autophagy to promote intracellular survival. During *Shigella* infection, septins assemble in certain pathways of actin polymerization. In turn, septins may control the shape of autophagic membranes [80]. Septins influence actin filament curvature [80,81] and may have a key role in biasing branched actin networks to mediate bacterial autophagy [68].

Recently, Barve et al. unbiasedly screened mutants with autophagy defects for the first time among conditionally lethal mutants in yeast. Upon the induction of autophagy, preassembled septin complexes relocate to the pre-autophagosomal structure (PAS) and form atypical septin rings [25,82]. They found that Cdc10 and Cdc11 interact with Atg9 at the PAS site during autophagy prevalent conditions. Septins participate in regulating autophagy by participating in the biosynthesis of autophagosomes [25,82]. In addition, septins traffic between different cellular compartments such as the Golgi, mitochondria, endosomes, plasma membrane, and vacuolar membranes [25]. However, there is no research on the relationship between septins and autophagy in filamentous fungi. Through these studies, we deduce that septins may be involved in the autophagy organization and transportation in filamentous fungi. As cytoskeleton proteins, septins function as “scaffold” to recruit autophagy related proteins participation in autophagy organization. In addition, septins are possibly involved in the autophagy by regulating MoAtg9 transportation betweeen ER and PAS. However, whether septin cooperates with autophagy to affect pathogenicity needs further exploration (Figure 4).

## Functions of septins in host infection by pathogens

More and more studies have shown that septins play important roles in both human and plant fungal pathogens [83,84]. In the plant pathogenic fungi *M. oryzae*, there are five septins, four of which (Sep3, Sep4, Sep5 and Sep6) are homologous to *S. cerevisiae* septins (Cdc3, Cdc10, Cdc11 and Cdc12) [13]. Septin tagged with GFP fluorescent protein found that these septins could formed a 5.9-μm ring at the appressorium pore. The deletion any of MoSep3, MoSep4, MoSep5 or MoSep6 in *M. oryzae* causes mislocalization, indicating that septins function as complexs [13]. During maturation of the appressorium, once the turgor pressure threshold is reached, the heterologous septin ring is formed at the bottom of the appressorium. The septin ring scaffold F-actin readjusts the circular network at the base of the appressorium to repolarize the appressorium to form a penetration peg to invade the rice leaves [13]. The septin ring also acts as a diffusion barrier to ensure the localization of the inverse-bin-amphiphysin-RVS-domain (I-BAR) protein Rvs167 and the Wiskott-Aldrich syndrome protein (WASP) Las17. The BAR protein is involved in membrane curvature at the tip of the penetration peg, and the WASP is involved in the polymerization of F-actin [13]. In addition, in *M. oryzae*, septin mutants showed a markedly reduced pathogenicity due to their inability to penetrate leaves, suggesting that septins were necessary for the development of rice blast disease [13]. Recent studies have shown that intercellular infection by *M. oryzae* is mediated by the fungal mitogen-activated protein kinase (MAPK) MoPmk1 [85]. After inhibiting the expression of MoPmk1, the hyphae are trapped in the original cells and do not infect adjacent cells. In this case, septins accumulate at the cell wall contact points but do not form the hyphae of the septin “collar” intercellular filaments (intercellular junctions) required for infection [85]. In the wheat pathogen *F. graminerarum*, its core septins FgCdc3, FgCdc11 and FgCdc12 (but not FgCdc10) are essential for fungal development and pathogenicity. Knockout of FgCdc3, FgCdc11 or FgCdc12 results in growth, differentiation and morphological defects, and podocytes differentiate into bifurcated conidia with additional polar growth. The pathogenicity of the Δ*Fgcdc3*, Δ*Fgcdc11* and Δ*Fgcdc12* mutants was significantly reduced. In contrast, the Δ*Fgcdc10* mutant is similar in growth, morphology, and pathogenicity to wild type [86]. In *U. maydis*, although septins are not essential for initial plant infection, but that the septins mutants were still capable of forming and producing large-scale tumors and teliospores. Septins can assemble into different structures that coexist in a cell: bud neck collars, band-like structures at the growing tip and long septin fibers near the cell cortex that link the two poles. Two of the two structures at the bud neck and the bud tip are similar to other structures already described in fungi. However, the third structure, the fiber runninging from pole to pole, is less described. Septin-deletion mutants are affected in the later stages of smut development, with well-designed bipolar instead of monofilaments and redundant germ tubes from teliospores. These mutants also showed reduced toxicity to corn [87]. Recent studies have found that the disruption of Sep4 in the *Botrytis cinerea* strain completely blocks the formation of infection structures (IFS) and eliminates the virulence of the Δ*Bcsep4* mutant on uninjured hosts. During the formation of IFS, mutants lacking *SEP4* can normally produce reactive oxygen species (ROS). The strain containing the *SEP4* gene inhibits the production of ROS, which leads to the assembly disorder of Sep4 and the inability to form infection cushions. This indicates that the correct assembly of Sep4 regulated by ROS is necessary for the IFS formation and infection. In addition, the loss of Sep4 severely impaired the conidia of the mutant, the accumulation of melanin and chitin at the tip of the hyphae and the expansion of lesions on the damaged host, but it significantly promoted the elongation of the embryo and the production of sclerotia [88]. In human pathogen *C. albicans*, Δ*cdc10* and Δ*cdc11* septin mutants were defective for invasive growth and displayed attenuated virulence in the mouse [89]. In *C. neoformans*, Δ*cdc3* and Δ*cdc12* septin mutants exhibited significantly attenuated virulence in the *Galleria mellonella* [15]. *Aspergillus fumigatus* includes five septins: AspA, AspB, AspC, AspD, and AspE. ∆*aspA*, ∆*aspB*, or ∆*aspC* mutants showed hypervirulence in a *G. melonella* model of infection. Infection with the Δ*aspD* or Δ*aspE* mutants in a *G. mellonella* model of invasive aspergillosis showed no virulence difference when compared to the wild-type strain. ∆*aspB* mutant showed wild-type virulence in a murine model of invasive aspergillosis [90].

## Research prospects

**Figure 5.**
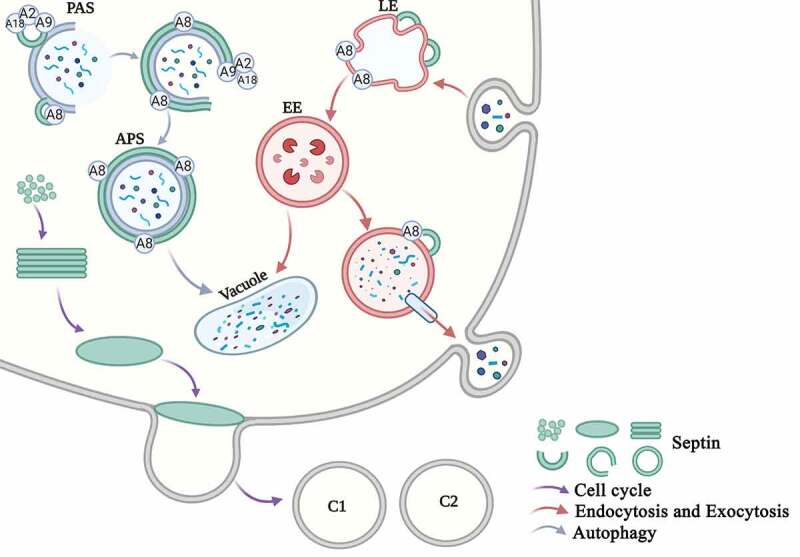
Overview of the main functions of septins in eukaryotic cells in *S. cerevisiae*. Cell cycle. Septins are mainly involved in the cell cycle progress. Free septins are recruited and aggregated into filaments, which then form a ring in the G1 phase and a collar in the S phase. The M-phase mother cell divides to form two daughter cells, and the septin ring also divides into the two daughter cells (C1 and C2). Endocytosis and Exocytosis. Septins are involved in the processes of endocytosis and exocytosis. The early endosomes swallow the extracellular proteins that enter the cell, and Sep3 and Sep6 bind to the early endosomes (EE). The late endosomes (LE) enter the vacuole and are degraded on the one hand, and they are secreted out of the cell through the vesicle on the other hand. The SNARE protein and the septin protein assists vesicle exocytosis. Autophagy. Septins are involved in autophagy. Septins are recruited to the PAS site. Sep4 interacts with Atg8 (A8), Atg9 (A9), and Atg18 (A18) and surrounds the autophagosomes (APS). Septins participate in the transport of Atg9 and the biosynthesis of the autophagosome

In the nearly 50 years of research since septins were first discovered, septins have been identified in many eukaryotic organisms. Septins interact with actin, microtubules, integrins and other cytoskeleton proteins and play important roles in important cellular and molecular pathways (Figure 5), thus providing many opportunities for future research.

Septins can be used as protein scaffolds to recruit proteins and as diffusion barriers to compartmentalize subcellular compartments. It is worth noting that these functions are not mutually exclusive, and may cooperate to mediate septin-dependent processes. In fungal pathogens, septins are not only related to their role in pathogenesis but also serve as diverse systems for understanding general septin biology. Among pathogenic fungi, the ability of septins to invade living host tissues appears to be critical. Therefore, the dynamics of septin assembly in living fungal cells requires further study during the infection process. We can use septin gene silencing or conditional allele methods to study whether the assembly of septin is directly inhibited during initial host infection or during later invasive growth. This requires the use of more advanced microscopes. Protein post-translational modifications (PTMs) increase the functional diversity of the proteins, proteolytic cleavage of regulatory subunits, or degradation of entire proteins. These modifications affect almost all aspects of normal cell biology and pathogenesis. During the assembly of septins into higher order structures, the direct or indirect action of kinases and phosphatases regulate the assembly of septins through phosphorylation/dephosphorylation. Although many studies are about how septin is modified during assembly and positioning [91–93], there are few studies on how septin is modified during fungi infection or autophagy process. So the role of the posttranslational modification of septin in fungi infection and autophagy process requires in-depth analysis. An important research direction is to systematically study the role of the posttranslational modification of septins in the dynamics of fungi infection and other cellular processes. In view of the key role of the posttranslational modifications of septins in host-pathogen interactions, the infection of plants by filamentous fungi may also help decipher the role of posttranslational modifications in regulating the assembly and function of septins. Diseases caused by autophagy defects include cancer, neurodegenerative diseases and so on [94–96]. Since septin dysfunction has been considered to be related to the pathogenesis of many of these diseases, it can be speculated that autophagy defects are the basis of the pathogenesis of certain septin diseases, and vice versa. The organization and precise function of septins in autophagy is expected to become an important area of future research. Given the general mechanism of autophagy from yeast to humans [97,98], it is unlikely that the interdependence between septins and autophagy is limited to bacterial autophagy. In mammals and yeast, septin interacts with SNARE protein. The biosynthesis of autophagosomes requires the fusion of SNARE, and actin can promote the fusion of autophagosomes to lysosomes. Therefore, septin may promote the fusion of membrane to autophagosomes and regulate the fusion of autophagosomes to lysosomes. It is important to study the role of septins in the assembly of various autophagy processes. Septins affect many human diseases, such as heart disease and cancer [99–102]. Future research on the biochemical and biological properties of septins will provide new insights into their functions, new biomarkers for disease diagnosis, and eventually new therapeutic targets for multiple diseases.

It is well known that *M. oryzae* is a model organism for studying autophagy in filamentous fungi. How septins coordinate and play roles in the autophagy process, how the septin ring is formed during the infection process, and how septins use the cell cycle to affect pathogenicity need to be studied. In this way, we can further discover the reasons that affect the pathogenicity and facilitate the further control of the rice blast fungus. The different complexes of septins have different functions in life processes, and how the septins precisely assemble in pathogenic fungi is still unknown. The crystal structure will be used to further reveal protein biological functions. In addition, the new chemical compounds based on the crystal structures of septins will provide a new method for the control of crop diseases. These research directions will be highly interesting.

## Data Availability

Data sharing not applicable-no new data generated. Data sharing is not applicable to this article as no new data were created or analyzed in this study.
